# Repair of post-laryngectomy pharyngocutaneous fistulas using a pectoralis major flap^☆^^☆☆^

**DOI:** 10.1016/j.bjorl.2018.03.002

**Published:** 2018-04-05

**Authors:** Anna Sumarroca, Elena Rodríguez-Bauzà, Joan Lop-Gros, Jacinto García, Montserrat López, Miquel Quer, Xavier León

**Affiliations:** a Universitat Autònoma de Barcelona, Hospital de la Santa Creu i Sant Pau, Otorhinolaryngology Department, Barcelona, Spain; b Universitat Autònoma de Barcelona, Hospital de la Santa Creu i Sant Pau, Plastic Surgery Department, Barcelona, Spain

**Keywords:** Total laryngectomy, Pharyngocutaneous fistula, Pectoralis major flap, Bypass salivary tube, Pharynx closure, Laringectomia total, Fístula faringocutânea, Retalho do músculo peitoral maior, Tubo de derivação salivar, Fechamento da faringe

## Abstract

**Introduction:**

The pectoralis major flap is a reconstructive option to consider in the treatment of pharyngocutaneous fistula after a total laryngectomy. There are not large studies assessing variables related to pharyngocutaneous fistula recurrence after removal of the larynx. Our objectives were to review the results obtained with this type of treatment when pharyngocutaneous fistula appears in laryngectomized patients, and to evaluate variables related to the results.

**Methods:**

We retrospectively reviewed our results using either a myocutaneous or fasciomuscular pectoralis major flap to repair pharyngocutaneous fistula in 50 patients.

**Results:**

There were no cases of flap necrosis. Oral intake after fistula repair with a pectoralis major flap was restored in 94% of cases. Fistula recurrence occurred in 22 cases (44%), and it was associated with a lengthening of the hospital stay. Performing the flap as an emergency procedure was associated with a significantly higher risk of fistula recurrence. Hospital stay was significantly shorter when a salivary tube was placed.

**Conclusions:**

The pectoralis major flap is a useful approach to repair pharyngocutaneous fistula. Placing salivary tubes during fistula repair significantly reduces hospital stay and complication severity in case of pharyngocutaneous fistula recurrence.

## Resumo

**Introdução:**

O retalho do músculo peitoral maior é uma opção a ser considerada no fechamento de fístula faringocutânea pós-laringectomia total. Não há grandes estudos que avaliem as variáveis relacionadas à recorrência da fístula faringocutânea após esse procedimento. Nossos objetivos foram avaliar os resultados obtidos com esse tipo de tratamento em pacientes laringectomizados com fístula faringocutânea e as variáveis relacionadas aos resultados.

**Método:**

Revisamos retrospectivamente os nossos resultados em 50 pacientes nos quais um retalho miocutâneo ou fasciomuscular do músculo peitoral maior foram utilizados para reparar a fístula faringocutânea.

**Resultados:**

Não houve casos de necrose de retalho. Após o reparo da fístula com um retalho do músculo peitoral maior, a ingestão oral foi restaurada em 94% dos casos. Houve recorrência da fístula em 22 casos (44%), a qual foi associada à duração da hospitalização. O uso do retalho como procedimento de emergência foi associado a um risco significativamente maior de recorrência da fístula. A permanência hospitalar foi significativamente menor quando utilizado um tubo de derivação salivar.

**Conclusões:**

O uso do retalho do músculo peitoral maior é uma abordagem útil para reparar a fístula faringocutânea. A colocação de tubos de derivação salivar durante o reparo da fístula reduz significativamente o tempo de hospitalização e a gravidade das complicações em caso de recorrência da fístula faringocutânea.

## Introduction

The appearance of a pharyngocutaneous fistula (PCF) remains one of the most common and difficult postoperative complications in patients undergoing total laryngectomy. This complication increases morbidity, length of hospitalization, and costs associated with treatment. The reported incidence of pharyngocutaneous fistulas after total laryngectomy is highly variable, ranging between 4% and 65%.^1^

Several studies have evaluated the risk factors associated with the appearance of PCF after total laryngectomy: previous treatment with radiotherapy^2^, ^3^, ^4^, ^5^, ^6^, ^7^, ^8^ or chemoradiotherapy^9^; a shorter interval between the end of radiotherapy and laryngectomy^7^, ^8^, ^10^; hemoglobin levels below 125 g/L preoperative^4^ as well as postoperative^5^, ^6^, ^11^; comorbidities such as diabetes, liver disease, or hypothyroidism^2^, ^3^, ^4^, ^12^; and surgical aspects such as neck dissection,^2^, ^7^, ^8^ prior tracheotomy,^6^ resection of the pharynx,^8^, ^13^ or the use of nonirradiated tissue to reinforce the pharyngeal suture.^14^ The results of a meta-analysis of 26 studies conducted by Paydafar et al.^15^ showed that risk factors significantly associated with the appearance of PCF were hemoglobin level less than 125 g/L, prior tracheostomy, preoperative radiotherapy, and concurrent neck dissection. The results also showed that PCF was more severe in patients with previous radiotherapy. In a case-control study conducted in laryngectomized patients by Venegas et al.,^16^ no significant differences were found in the percentage of PCF between non-irradiated and irradiated patients (12% vs. 18%, *p* > 0.05). However, patients with previous radiotherapy and PCF required more surgical repair procedures and the length of hospitalization was increased.

Treatment for PCF is usually conservative. It consists of medical treatment with antibiotics, enteral nutrition with a nasogastric tube, and daily local wound care including removal of all necrotic tissue, cleaning with an antiseptic solution, and placement of a compressive dressing. Between 62%^3^ and 86%^17^ of PCF spontaneously close with medical treatment, particularly those in non-irradiated patients with small fistulas. Surgical closure of the fistula is indicated when conservative treatment fails, but there is no consensus about waiting time. Several authors consider there is no reason to wait any longer if PCF closure is not obtained in one month with medical management; surgical closure should be considered in these patients.^2^, ^11^, ^18^

Many surgical techniques are used in the treatment of the PCF: direct closure^2^, ^11^; endoscopic repair with a platysmal flap^19^; axial fasciocutaneous flaps such as the deltopectoral flap^18^, ^20^ or the internal mammary artery perforator flap^21^; musculocutaneous flaps such as the sternocleidomastoid,^22^ pectoralis major flap,^18^, ^20^, ^23^, ^24^, ^25^ trapezius^26^ or latissimus dorsi^22^ flaps; and free flaps such as the jejunum,^23^, ^27^ radial forearm^5^, ^12^, ^25^, ^28^ and anterolateral thigh flaps.^29^

In our center, the method of choice to treat a PCF that does not respond adequately to conservative treatment is fistula closure using a pectoralis major flap (PMF). Since 1994 we have systematically added placement of a bypass salivary tube (Montgomery^®^ Salivary Bypass Tube; Boston Medical Products) in combination with the PMF.

The aims of this study were to review the results obtained with this type of treatment when PCF appears in laryngectomized patients and to evaluate variables related to the results.

## Methods

Data were obtained retrospectively from a registry that prospectively collects information from reconstructive procedures performed in our center since 1990. From 1990 to 2014, a total of 50 patients were treated with a PMF to close post-laryngectomy PCF. Table 1 shows the characteristics of patients included in this study.

**Table 1:** Characteristics of patients included in the study (*n* = 50).

*Age, years (average, standard deviation)*	61.4 (9.1)
*Sex*	
Male	47 (94%)
Female	3 (6%)

*Location*	
Larynx	35 (70%)
Hypopharynx	15 (30%)

*Prior radiotherapy*	
No	11 (22%)
Yes	39 (78%)

*Previous flap*	
No	44 (88%)
Yes	6 (12%)

*Preoperative Hb g/L (average, standard deviation)*	109.9 (18.7)
*Fistula period in days (average, range)*	32 (5–300)

In one case, the fistula appeared due to necrosis of a radial free skin flap used in reconstruction of the hypopharynx. In two patients a PMF was used after the previous failure of a repair attempt with an internal mammary artery perforator flap. In six patients treated with a total laryngectomy and a partial or total pharyngectomy a PMF was used in the reconstruction of the hypopharynx at the primary surgery.

The decision for elective surgical closure of PCF with a PMF was carried out after a failed conservative management of at least four weeks duration and was based on clinical parameters such fistula size and cervical soft tissue status.

Treatment involved the use of a myocutaneous or fasciomuscular flaps. The criteria to use a myocutaneous flap or a fasciomuscular flap depended basically on the size of the defect to be reconstructed and the morphology of the patient. We chose a muscle flap without skin paddle when fistula repair required reconstruction of less than a third of the hypopharynx, or when the thickness of the skin paddle was considered excessive. In these cases, we directly sutured the superficial fascia of the pectoralis muscle to the defect margins. In the case of large pharyngocutaneous fistulas requiring reconstruction of more than a third of the hypopharynx, we used myocutaneous flaps, suturing the skin paddle to the margins of the pharyngeal defect. Since 1994, we routinely place a bypass salivary tube simultaneously with pharyngocutaneous fistula repair.

Full oral intake restoration after PCF repair using the PMF was considered as a successful outcome, as long as no new further PCF repair procedures were needed. Analysis of the results included the percentage of complications at the cervical surgical wound site and thoracic donor site, and the period of hospitalization after performing the pectoralis flap repair.

Qualitative variables were compared using the Chi-square test or Fisher's exact test. The relationship between qualitative and continuous variables was performed using the nonparametric Mann–Whitney test. A logistic regression was used in the multivariate analysis.

This study was approved by the institutional oncological scientific committee of our center, and conducted in accordance with the Declaration of Helsinki.

## Results

A pectoralis myocutaneous flap was performed in 12 cases (24.0%), and a fasciomuscular flap without a skin paddle in 38 cases (76.0%). No signs of flap necrosis were observed in any case.

The flap was performed within 14 days post-laryngectomy in 13 patients with a pharyngocutaneous fistula and cervical bleeding. In these cases, the flap was used in an emergency context to protect the vascular axis and to reconstruct the pharyngeal defect, performing the suture on a contaminated area. In the remaining patients, the fistula was repaired electively, and no evidence of cervical wound infection was noted during surgery.

One patient with a PCF had a hemorrhage of the internal jugular vein on the 12th postoperative day. Following vein ligation we repaired the fistula using a myocutaneous pectoralis flap. The patient died eight days later due to septic shock associated with complications of the cervical wound; the fistula persisted at the time of death. Two patients required a second surgery to repair the recurrence of PCF after treatment with the PMF. In the two cases, surgical treatment consisted of the use of a second PMF to repair the defect.

Restoration of full oral intake is considered the goal of treatment. Post-laryngectomy PCF surgical repair using CMPM was successful in 94% of patients (47/50). PCF recurrence occurred in 22 of the 50 procedures (44%). Table 2 shows the percentage of patients with fistula recurrence in relation to patient age, hemoglobin levels, the number of days before repair, the location of the primary tumor, previous radiotherapy, the use of PMF at the primary surgery, the type of flap, the use of a salivary bypass tube, and the context of the repair surgery (elective or emergency with a cervical bleeding). There were no significant differences in fistula recurrence after completion of the pectoralis flap for any of the analyzed variables. We found a correlation for fistula recurrence when surgery was carried out as an emergency procedure. Two of the patients operated in an emergency situation needed a second reconstructive procedure with a contralateral PMF in order to a close recurrent PCF.

**Table 2:** Fistula recurrence after repair with a pectoral flap in relation to clinical and operative variables.

	Fistula recurrence		*p*
	No	Yes	
*Age in years (median)*	60.8	62.7	0.453
*Period of previous fistula days (median)*	32.0	33.0	0.692
*Preoperative Hb in g/L (median)*	114.1	106.0	0.140
*Location*			
Larynx	21 (57%)	16 (43%)	0.757
Hypopharynx	8 (53%)	7 (47%)	

*Radiotherapy*			
No	6 (55%)	5 (45%)	0.927
Yes	23 (56%)	18 (44%)	

*Previous flap*			
No	26 (57%)	20 (43%)	0.762
Yes	3 (50%)	3 (50%)	

*Flap*			
Myocutaneous	8 (67%)	4 (33%)	0.386
Fasciocutaneous	21 (53%)	19 (47%)	

*Salivary*			
No	8 (42%)	11 (58%)	0.132
Yes	21 (64%)	12 (36%)	

*Emergency*			
No	25 (64%)	14 (37%)	0.054
Yes	4 (31%)	9 (69%)	

We performed a multivariate study considering fistula recurrence as the dependent variable and including those variables with a *p* < 0.20 in the univariate analysis as the independent variables (Table 3). The only variable associated with a significant risk of fistula recurrence was undergoing flap surgery as an emergency procedure. In this situation, the risk of fistula recurrence was 4.68 times higher (95% CI 1.05–20.83, *p* = 0.043). Patients in whom salivary tube bypass was not used had an increased risk of recurrence, but in this case the hazard ratio did not reach statistical significance (HR = 3.42, 95% CI 0.94–12.37, *p* = 0.060).

**Table 3:** Results of multivariate analysis considering fistula recurrence as a dependent variable.

	HR	95% CI	*p*
*Salivary*			
Yes	1		
No	3.42	0.94–12.37	0.060

*Emergency*			
No	1		
Yes	4.68	1.05–20.83	0.043

*Preoperative Hb*	0.984	0.951–1.01	0.356

**Figure 1:**
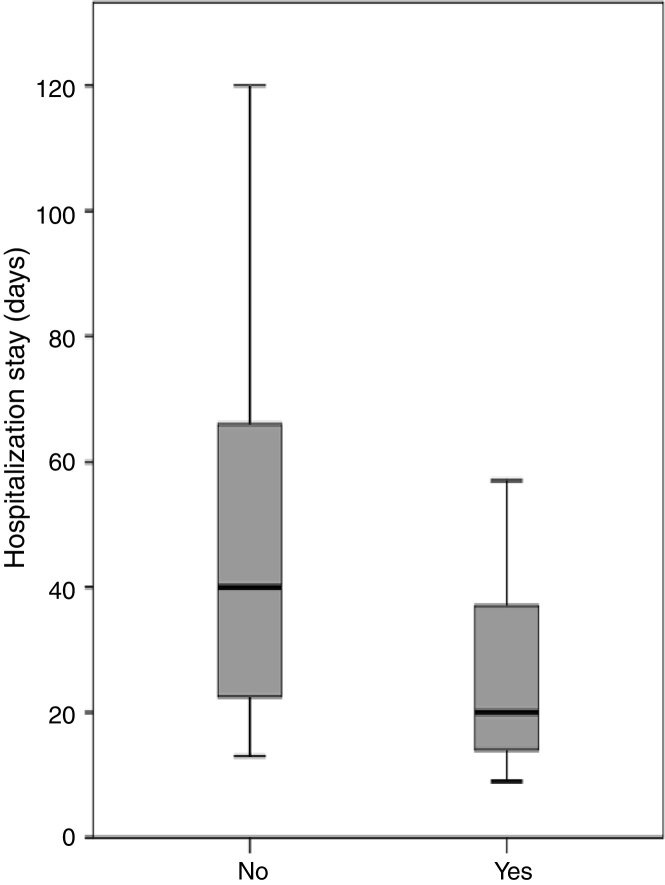
Length of hospitalization stay in days in relation to the use of salivary bypass tube.

Three patients were discharged from hospital with a nasogastric tube due to the persistence of a small fistula that was resolved on an outpatient basis. With the exception of the patient who died during the postoperative period, all patients achieved successful oral intake. The median length of hospitalization after completion of the pectoralis flap was 24.5 days (range 9–120 days). Length of hospitalization after completion of a pectoralis flap differed significantly depending on fistula recurrence (*p* = 0.001). The median hospitalization for patients who were treated with the pectoralis flap was 16.0 days (range 9–31 days) for those who achieved primary closure of the fistula, and 45 days (range 13–120 days) for patients with fistula recurrence. Periods of hospitalization after completion of the PMF were analyzed considering the location of the primary tumor, previous radiotherapy, type of flap, the use of a shunt salivary tube, and whether the flap was performed as elective or emergency surgery. Placement of a salivary bypass tube was the only variable significantly related to the length of stay (Fig. 1). The median hospitalization was 20.5 days (range 9–90 days) for patients in whom a salivary tube was placed, and 40.0 days (range 13–120) for patients in whom no tube was placed (*p* = 0.013). Considering only patients with fistula recurrence (*n* = 22), the median length of hospitalization was 41.5 days (range 13–90 days) for patients in whom a salivary tube was placed (*n* = 12) and 66 days (range 40–120 days) for patients in whom no tube was placed (*n* = 10) (*p* = 0.036).

Five patients (9.6%) had a chest wound infection in the donor area and 2 (3.8%) had a hematoma. All 7 cases of chest level complication resolved with local wound care.

## Discussion

Several studies have shown the efficiency of the PMF in closing the pharynx during a laryngectomy and hence reducing the frequency of postoperative PCF.^30^, ^31^ However, to our knowledge no studies have been performed in a large series of patients to examine the usefulness of PMF to treat postoperative PCFs or to determine the variables related to the appearance of fistula recurrence.

According to our results, fasciomuscular and myocutaneous pectoralis flaps for the closure of post-laryngectomy PCF are safe and effective. With the exception of the patient who died due to complicated postoperative wound infection and secondary septic shock, oral intake was restored in all patients, although a second procedure using contralateral PMF was needed in two of them. Despite the usefulness and efficacy of this technique, 44% of patients developed fistula recurrence. Performing this surgery in an emergency situation due to postoperative local bleeding was the variable most strongly associated with the recurrence of PCF, with a 67% rate of fistula recurrence. In contrast, when surgery was performed as elective non-emergency surgery, the frequency of fistula recurrence was 37%, a rate similar to that reported by other authors. McLean et al.^23^ analyzed the results obtained in 17 cases of post-laryngectomy PCF repaired with PMF in a group of patients in which 82.4% had received pre-operative irradiation. These patients had 58.8% of complications associated with PMF reconstruction, with a 35.3% rate of fistula recurrence. In Magdy et al.^25^ study, the rate of fistula recurrence in 10 patients using PMF to repair post-laryngectomy PCF was 30%.

The results of a multivariate analysis showed that the use of the PMF in an emergency situation increased the risk of fistula recurrence almost nine-fold. In our opinion, the increased risk of fistula recurrence when PMF reconstruction is carried out in an emergency background is justified by the precarious systemic and local context in which surgery is performed. When cervical bleeding is associated with pharynx and cervical dehiscence the pectoralis flap is sutured on infected tissue. Cervical bleeding means, furthermore, that we are working on hemodynamically unstable patients. Given these results, unless a flap is required to protect the vascular axis, in the emergency situation we now prefer to perform a pharyngostoma, suturing the margins of the pharyngeal dehiscence to cervical skin, and to defer the reconstruction of the PCF with a PMF until local and systemic conditions improve.

In an attempt to reduce the incidence of fistula recurrence, since 1994 we have been systematically placing a salivary bypass tube during PCF closure. Our results showed a trend to reduce the percentage of fistula recurrence (56% in cases without a bypass tube versus 37% in cases with a bypass tube), the differences not reaching statistical significance. However, we observed a significant decrease in the length of hospitalization in patients with a salivary bypass tube, even in patients with fistula recurrence.

The main advantages in using the pectoralis flap in the repair of PCF are that it is a technically simple procedure, and that it provides a well-vascularized tissue with a pedicle that easily reaches the cervical region. Disadvantages of this flap are the morbidity caused at the donor site, and excessive bulk depending on the phenotype of the patient, especially in cases where a myocutaneous flap is used. It should be kept in mind, however, that as all our patients were operated by the same surgical team, the learning curve using the PMF in the closure of PCF could have played a role in the better results achieved over time.

An alternative to pectoralis flaps are microanastomosed free flaps.^27^ The advantages of this type of repair are the availability of different donor sites and their adaptability to the specific requirements of each type of defect. Free flaps, however, also have disadvantages. These include increased complexity of the microsurgical technique and problems arising from a lack of viable vascular neck structures in patients with a history of neck dissection, radiotherapy, or cervical infection. In the series of Bohannon and cols^27^ in which free flaps were used to repair 20 cases of PCF, the authors chose to use recipient vessels from areas other than the neck in 45.5% of cases, anastomosing the free flap to the internal mammary vessels.

## Conclusions

The myocutaneous or fasciomuscular PMF is a useful reconstructive tool for the repair of PCFs after laryngectomy. Placing salivary by-pass tubes during fistula repair significantly reduces hospital stay and the severity of complications of PCF recurrence.

## Conflicts of interest

The authors declare no conflicts of interest.
